# Development trajectories of marital satisfaction and postpartum depression: a longitudinal study

**DOI:** 10.3389/fmed.2026.1837652

**Published:** 2026-06-12

**Authors:** Yunjuan Ji, Liping Chen, Xiang Shi

**Affiliations:** Department of Obstetrics, The First People’s Hospital of Nantong, Nantong, Jiangsu, China

**Keywords:** cross-lagged model, latent growth model, longitudinal study, marital satisfaction, postpartum depression

## Abstract

**Objective:**

This study aimed to utilize a longitudinal design to delineate the developmental trajectories of marital satisfaction and depressive symptoms in the early postpartum period, examine their longitudinal predictive relationship to examine the directionality, and explore the moderating effects of demographic and obstetric factors on this relationship.

**Methods:**

A convenience sampling method was employed to recruit 238 postpartum women from Nantong First People’s Hospital between February and August 2025. Participants were assessed at three time points: 1, 6, and 12 weeks postpartum. Data were collected using the Marital Satisfaction Scale and the Edinburgh Postnatal Depression Scale. Data analysis was performed using SPSS 26.0 for descriptive statistics and correlation analysis. Mplus 8.0 was used to construct Latent Growth Models and a Cross-Lagged Panel Model to analyze developmental trajectories and bidirectional predictive relationships.

**Results:**

Developmental Trajectories: Marital satisfaction showed a significant linear declining trend over the 12-week postpartum period; based on linear growth model fitting, postpartum depressive symptoms also exhibited a significant linear increasing trend. Predictive Relationship: Cross-lagged model analysis revealed that earlier marital satisfaction consistently and negatively predicted later levels of depression. However, no significant longitudinal predictive effect of depressive symptoms on marital satisfaction was found, supporting a unidirectional predictive pattern from marital satisfaction to postpartum depression. Moderating Effects: The protective association of marital satisfaction was significantly stronger among unemployed/housewives, those with low monthly household income and those who underwent cesarean section.

**Conclusion:**

This study demonstrates that marital satisfaction is a significant longitudinal protective factor associated with lower levels of postpartum depression, and the strength of this association is moderated by individual socioeconomic resources and health challenges. The findings support integrating the assessment and intervention of marital relationships into postpartum mental healthcare and provide empirical evidence for implementing precise, couple-centered prevention strategies targeting high-risk populations.

## 1 Introduction

Amid adjustments to family planning policies and the accelerated pace of modern life, Postpartum Depression (PPD) has emerged as a significant public health concern. As the most common psychiatric disorder in the perinatal period, the prevalence of PPD remains persistently high globally and domestically, with studies in China reporting an incidence of approximately 22.14% (1). PPD poses a serious threat to the wellbeing of mothers, infants, and families. The condition, characterized by persistent low mood, loss of interest, anxiety, and exhaustion, can also impair maternal cognitive function, diminish self-worth, and severely compromise social and parenting abilities (2, 3). More profoundly, by negatively affecting the quality of mother-infant interaction, PPD exerts significant detrimental effects on the infant’s emotional, cognitive, and behavioral development (4, 5). Therefore, investigating the key influencing factors and dynamic developmental patterns of PPD is of crucial public health significance for formulating effective prevention and intervention strategies.

The postpartum period is a critical stage marked by significant physiological, psychological, and social role transitions for women. During this time, the marital relationship, serving as the mother’s most core and intimate source of social support, is widely considered a decisive factor influencing her psychological adaptation (6). Marital Satisfaction (MS) (7), defined as an individual’s subjective positive evaluation and emotional experience of the marital relationship, faces severe challenges during this phase. A sharp increase in parenting stress, sleep deprivation, changes in sexual intimacy, and shifts in family focus can all strain spousal interaction patterns and relationship quality, leading to significant fluctuations in marital satisfaction in the early postpartum period (8). Cross-sectional studies consistently demonstrate a significant negative correlation between marital satisfaction and postpartum depressive symptoms (9, 10).

However, existing research, primarily based on cross-sectional designs, struggles to elucidate the causal direction and dynamic interplay between these two constructs. Based on the Spillover Effect (11) and Family Systems Theory (12), marital satisfaction and postpartum depression likely form a bidirectional, mutually reinforcing vicious cycle. On one hand, low marital satisfaction, potentially leading to insufficient spousal support and increased conflict, may act as a key risk factor for triggering or exacerbating postpartum depression (the social support buffering model). On the other hand, depressive symptoms exhibited by the mother, such as emotional instability, withdrawal, and loss of interest, can adversely affect marital intimacy, effective communication, and cooperative parenting, thereby further eroding marital quality and creating a negative feedback loop of “depression → marital dissatisfaction → more depression.” Currently, the dynamic evolution of this bidirectional relationship remains poorly understood. Does a decline in marital satisfaction precede and predict the onset of postpartum depression, or do depressive symptoms precede and lead to reduced marital satisfaction? What are the developmental trajectories of both constructs over the first few postpartum months? How does the strength of their mutual predictive effects vary across different time points? These critical questions cannot be adequately answered by cross-sectional studies alone.

To address these gaps, this study employs a prospective longitudinal design with repeated measurements at three time points: 1 week (T1), 6 weeks (T2), and 12 weeks (T3) postpartum. The primary aims of this study are: (1) to delineate the dynamic developmental trajectories of maternal marital satisfaction and postpartum depressive symptoms from the early postpartum period to 3 months postpartum; (2) to examine the predictive effect of early marital satisfaction on later postpartum depression levels, and simultaneously, the predictive effect of early postpartum depressive symptoms on later marital satisfaction, thereby examining the possibility of a bidirectional predictive relationship; and (3) to explore the moderating effects of demographic and obstetric factors on this dynamic relationship. By adopting a developmental perspective, this research seeks to examine the directionality and potential temporal ordering between marital satisfaction and postpartum depression. The findings aim to provide precise empirical evidence to assist clinicians in early identification of at-risk populations during the critical postpartum period and in developing targeted psychological interventions focused on the couple’s relationship. The ultimate goal is to mitigate potential negative cycles and enhance maternal mental health and marital quality.

## 2 Participants and methods

### 2.1 Participants

A convenience sampling method was used to recruit and follow up with pregnant women who received regular prenatal care and delivered at the Department of Obstetrics, Nantong First People’s Hospital, from February 2025 to August 2025.

Inclusion criteria: ➀Age ≥20 years; ➁Singleton, full-term pregnancy (gestational age ≥37 weeks); ➂Underwent regular prenatal examinations and delivered at the study hospital; ➃Provided informed consent and voluntarily participated in the study.

Exclusion criteria: ➀Presence of severe pregnancy complications or comorbidities, such as severe preeclampsia, gestational diabetes requiring insulin therapy, placental abruption, etc.; ➁History of mental disorders or family history of mental illness; ➂Infant admitted to the Neonatal Intensive Care Unit (NICU) after birth; ➃Presence of severe communication barriers or inability to comprehend the questionnaire content.

Withdrawal and loss-to-follow-up criteria: ➀Participants voluntarily withdrew from the study for personal reasons; ➁Loss to follow-up due to relocation, change of contact information, etc.; ➂Missing data from any of the three questionnaire surveys.

Sample size calculation: The sample size was estimated using G*Power 3.1 software. A repeated measures ANOVA (within-between interaction) was selected, with a medium effect size (*f* = 0.25), α error probability of 0.05, statistical power (1-β) of 0.95, and the number of groups (postpartum time points) set to 3. The calculated minimum total sample size was 216. Considering an approximate 15% attrition rate common in longitudinal studies, the initial target sample size was set to 250. Ultimately, 238 participants completed all three follow-up surveys. This study protocol was approved by the Ethics Committee of Nantong First People’s Hospital (Approval No.: 2025KT153).

### 2.2 Methods

#### 2.2.1 Measurement instruments

(1) General Information Questionnaire: Designed by the researchers, it collected socio-demographic and obstetric data, including maternal age, education level, occupation, monthly household income, mode of delivery, and infant sex.

(2) Marital Satisfaction Scale (MSS): The marital satisfaction subscale (short version) from Olson’s Marital Quality Questionnaire was used (13). This scale consists of 10 items rated on a 5-point Likert scale from 1 (“strongly disagree”) to 5 (“strongly agree”). Items Q1, Q3, Q5, Q8, and Q9 are reverse-scored. The total score ranges from 10 to 50, with higher scores indicating greater marital satisfaction. In this study, the scale demonstrated a Cronbach’s α coefficient of 0.89.

(3) Edinburgh Postnatal Depression Scale (EPDS): The Chinese version of the scale, developed by Cox (14) and translated by Wang Ying et al., was used. The scale comprises 10 items, each rated on a 4-point scale (0–3). The total score ranges from 0 to 30, with higher scores indicating more severe depressive symptoms. An EPDS total score >10 was used as the cutoff for screening postpartum depression. In this study, the scale demonstrated a Cronbach’s α coefficient of 0.86.

#### 2.2.2 Data collection procedure

Drawing on the design of previous longitudinal studies and considering the trajectory of postpartum psychological adjustment, the study aims, and available resources, three assessment time points were determined: 1 week postpartum (T1, baseline), 6 weeks postpartum (T2), and 12 weeks postpartum (T3).

The T1 (baseline) assessment was conducted during the participant’s hospital stay or at their first postpartum hospital visit. The principal investigator and two trained obstetric nurses administered the questionnaires in the obstetric wards or outpatient clinics of Nantong First People’s Hospital. Before the assessment, researchers explained the study purpose, significance, questionnaire instructions, and privacy protection measures in detail to establish rapport, secure long-term cooperation, and obtain written informed consent. Participants completed the questionnaires independently. For any unclear items, investigators provided neutral explanations, avoiding leading suggestions. All questionnaires were checked and collected on-site to ensure completeness. The T2 and T3 follow-up assessments were conducted primarily using online methods. 1 week before each assessment, researchers sent reminders via phone or WeChat to inform participants of the upcoming survey. When the assessment was due, a professional online survey platform was used to generate links or QR codes, which were sent to participants for online self-administration. For non-respondents, researchers made phone call reminders. If necessary, questionnaires were completed via item-by-item telephone inquiry by the researcher. Upon electronic submission, the system automatically recorded the submission time. Researchers promptly reviewed data quality. If logical inconsistencies or response patterns (e.g., selecting the same option for all items) were detected, confirmation was sought from participants via phone to ensure data authenticity and validity.

In this study, 250, 242, and 238 valid questionnaires were successfully collected at T1, T2, and T3, respectively. During follow-up, 12 participants were excluded due to loss of contact (changed contact information, unresponsive to multiple attempts), voluntary withdrawal, or incomplete data across all three time points. Ultimately, 238 participants completed all three follow-up assessments, resulting in a valid follow-up rate of 95.2%.

#### 2.2.3 Statistical analysis

Statistical analysis was performed using SPSS 26.0 for data management and descriptive statistics. Continuous variables are presented as mean ± standard deviation ($\bar{x}$ ± s), and categorical variables as frequencies and percentages (%). Pearson correlation analysis was employed to examine the bivariate relationships between marital satisfaction and postpartum depression at each time point.

To conduct in-depth analysis of the longitudinal data, Mplus 8.0 software was utilized to construct the following models: First, unconditional linear Latent Growth Models (LGMs) were constructed separately for marital satisfaction and postpartum depression to examine their developmental trajectories—including initial levels and rates of change—across the three time points: 1 week (T1), 6 weeks (T2), and 12 weeks (T3) postpartum. Subsequently, building upon the univariate models, a Parallel Process Latent Growth Model was constructed to investigate the association between the developmental trajectories of marital satisfaction and postpartum depression from a developmental perspective. Finally, a Cross-Lagged Panel Model (CLPM) was constructed to test the predictive effects of T1 marital satisfaction on T2 postpartum depression and T2 marital satisfaction on T3 postpartum depression, as well as the predictive effects of T1 postpartum depression on T2 marital satisfaction and T2 postpartum depression on T3 marital satisfaction, thereby exploring their potential mutual predictive relationships. Model parameters were estimated using the Maximum Likelihood Robust Estimator (MLR), which effectively handles non-normally distributed data and provides correct standard error estimates. The models were evaluated and the optimal model was determined based on a comprehensive assessment of fit indices (χ^2^, CFI, TLI, RMSEA, and SRMR) combined with the results of nested model chi-square difference tests. A two-sided *p*-value < 0.05 was considered statistically significant for all analyses.

## 3 Results

### 3.1 Results of the general information questionnaire

A total of 238 postpartum women were ultimately included in the analysis. The mean age of the participants was 28.71 ± 3.85 years. The sociodemographic and obstetric characteristics of the study sample are detailed in Table 1.

**TABLE 1:** General characteristics of the study participants (*N* = 238).

Characteristic	Category	Number (n)	Proportion (%)
Age (years)		28.71 ± 3.85*	
	≤ 25	47	19.7
	26–30	128	53.8
	31–35	52	21.8
	> 35	11	4.6
Education level	High school/Technical secondary school and below	65	27.3
	College/Bachelor’s degree	160	67.2
	Master’s degree or above	13	5.5
Employment status	Employed	156	65.5
	Unemployed/Housewife	82	34.5
Monthly household income per capita (RMB)	< 5,000	31	13.0
	5,000–10,000	127	53.4
	>10,000	80	33.6
Mode of delivery	Vaginal delivery	138	58.0
	Cesarean section	100	42.0
Infant sex	Male	122	51.3
	Female	116	48.7

*Mean ± standard deviation.

### 3.2 Common method bias test

As the data for the core variables (marital satisfaction and postpartum depression) in this study were derived from self-reports by the postpartum women, common method bias was a potential concern. To assess the severity of this bias, Harman’s single-factor test was conducted separately on the data from the three survey waves (T1, T2, T3). The results indicated that for the T1, T2, and T3 data, the numbers of factors with eigenvalues greater than 1 were 12, 10, and 11, respectively. The variance explained by the first factor was 24.872, 26.119, and 28.335%, respectively, all of which were well below the critical threshold of 40%. This suggests that no single factor accounted for the majority of the variance in the data from any of the three surveys, indicating the absence of severe common method bias in this study.

### 3.3 Scores and correlation analysis of marital satisfaction and postpartum depression

**FIGURE 1:**
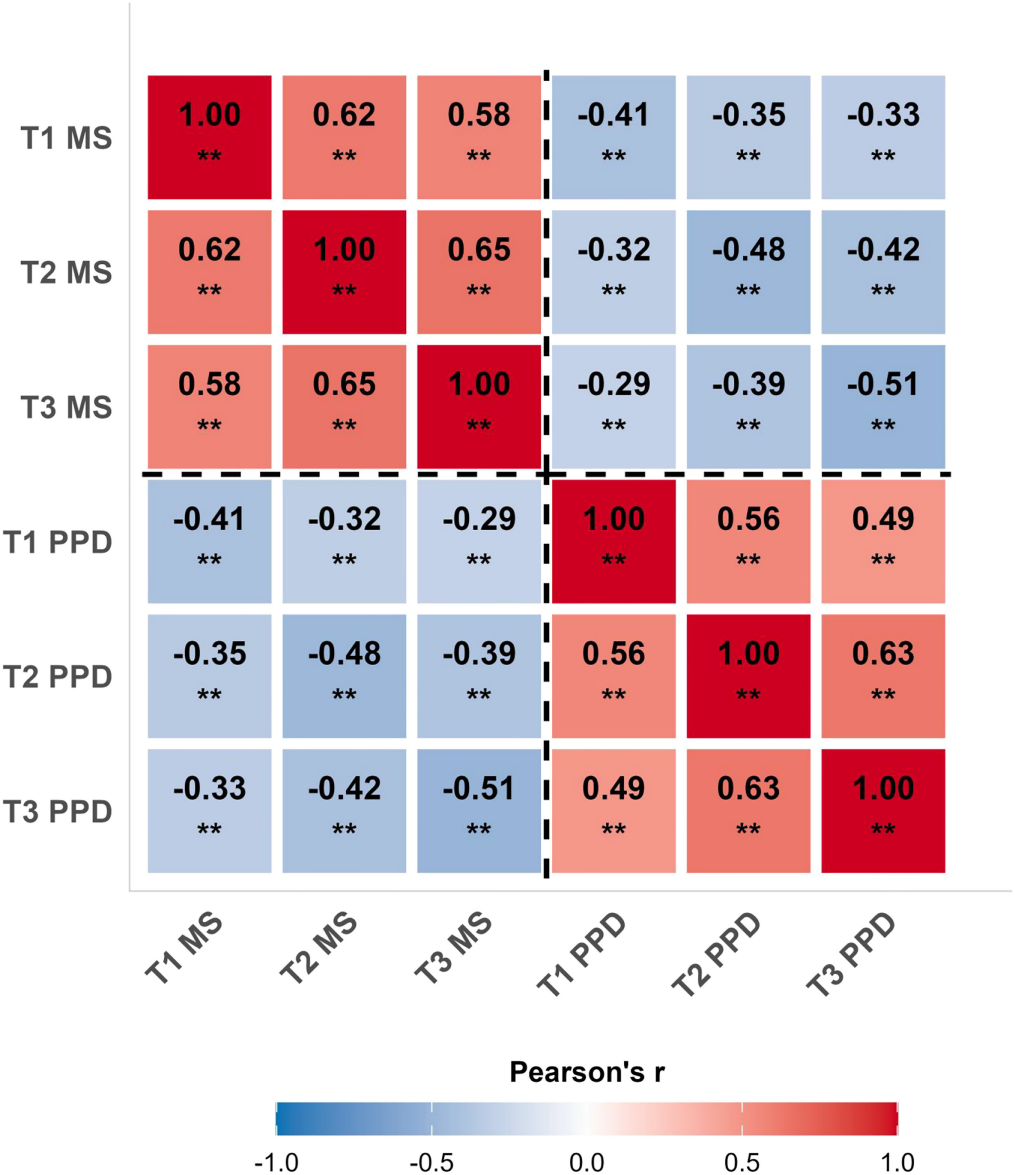
Analysis of the correlation between maternal marital satisfaction and postpartum depression.

The total scores for marital satisfaction across the three time points were (38.24 ± 5.62), (36.85 ± 6.13), and (37.02 ± 5.89). The total scores for postpartum depression were (7.85 ± 3.42), (9.23 ± 4.01), and (8.67 ± 3.76). Correlation analysis results (see Table 2 and Figure 1) indicated that marital satisfaction and postpartum depression showed significant negative correlations at all-time points (*P* < 0.01).

**TABLE 2:** Correlation analysis of marital satisfaction and postpartum depression (r values).

Variable	MS-T1	MS-T2	MS-T3	PPD-T1	PPD-T1	PPD-T3
MS-T1	1	1	1	1	1	1
MS-T2	0.62**					
MS-T3	0.58**	0.65**				
PPD-T1	−0.41**	−0.32**	−0.29**			
PPD-T1	−0.35**	−0.48**	−0.39**	0.56**		
PPD-T3	−0.33**	−0.42**	−0.51**	0.49**	0.63**	

**p < 0.01; MS, Marital Satisfaction; PPD, Postpartum Depression; T1, 1 week postpartum; T2, 6 weeks postpartum; T3, 12 weeks postpartum.

### 3.4 Parallel process latent growth model of marital satisfaction and postpartum depression

A linear Latent Growth Model (LGM) was employed to analyze the developmental trajectories and the interrelationship between marital satisfaction and postpartum depression. The model comprises two latent variables: the intercept factor (I), representing the initial level of a variable, and the slope factor (S), representing its rate of change. Since the three measurement time points were 1 week (T1), 6 weeks (T2), and 12 weeks (T3) postpartum, with intervals of 5 and 6 weeks, the factor loadings for the slope were set to 0, 1, and 2.2, respectively, based on the actual proportion of weeks elapsed (T1→T2 = 5 weeks, T1→T3 = 11 weeks; thus, T2 loading = 5/5 = 1, T3 loading = 11/5 = 2.2).

#### 3.4.1 Developmental trajectory of marital satisfaction

**FIGURE 2:**
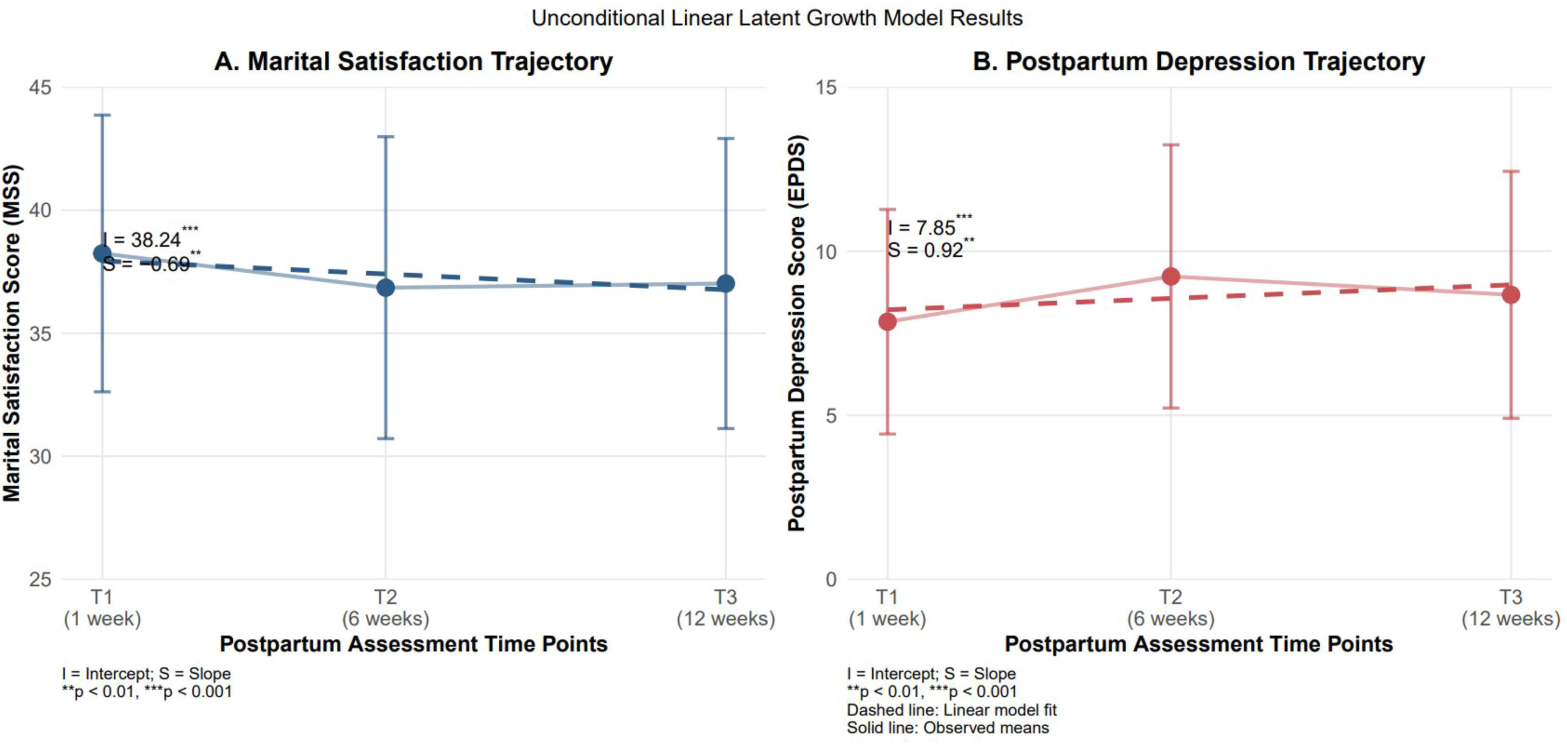
Developmental trajectories of marital satisfaction and postpartum depression. **(A)** Marital satisfaction (MSS) shows a linear declining trend. **(B)** Postpartum depression (EPDS) shows a linear increasing trend based on latent growth modeling. Error bars represent ± 1 SD. Dashed lines represent linear model fits. ***p < 0.001, **p < 0.01.

An unconditional linear LGM for marital satisfaction was constructed. The fit indices were excellent: χ^2^/df = 2.215, CFI = 0.996, TLI = 0.989, RMSEA = 0.072, SRMR = 0.019. The intercept factor, representing the initial level of marital satisfaction, was 38.24 (*P* < 0.001). The linear slope was −0.69 (*P* = 0.008), indicating a significant overall linear decline in marital satisfaction across the three measurements. Both the intercept variance (σ^2^ = 31.58, *P* < 0.001) and slope variance (σ^2^ = 2.87, *P* = 0.026) were significant, suggesting significant inter-individual differences in the initial level and the rate of change in marital satisfaction. The intercept and slope factors were significantly negatively correlated (*r* = –0.682, *P* < 0.001), indicating that a higher initial level of marital satisfaction was associated with a faster subsequent rate of decline (see Figure 2).

#### 3.4.2 Developmental trajectory of postpartum depression

An unconditional linear LGM for postpartum depression was constructed. The fit indices were acceptable: χ^2^/df = 2.895, CFI = 0.993, TLI = 0.985, RMSEA = 0.089, SRMR = 0.023. The intercept factor, representing the initial level of postpartum depression, was 7.85 (*P* < 0.001). The linear slope was 0.92 (*P* = 0.005), indicating a significant linear increase in depressive symptoms during the measurement period under the linear growth assumption. However, the observed means (7.85 ± 3.42, 9.23 ± 4.01, 8.67 ± 3.76) suggest that postpartum depressive symptoms may have peaked around 6 weeks postpartum before slightly declining, implying a potential non-linear pattern that the linear model may not fully capture. The intercept variance was significant (σ^2^ = 11.73, *P* < 0.001), but the slope variance was not (σ^2^ = 1.42, *P* = 0.107), indicating significant inter-individual differences in the initial depression level but not in the linear rate of change. The correlation between the intercept and slope was not significant (*r* = 0.508, *P* = 0.134) (see Figure 2).

#### 3.4.3 Dynamic relationship between marital satisfaction and postpartum depression

**FIGURE 3:**
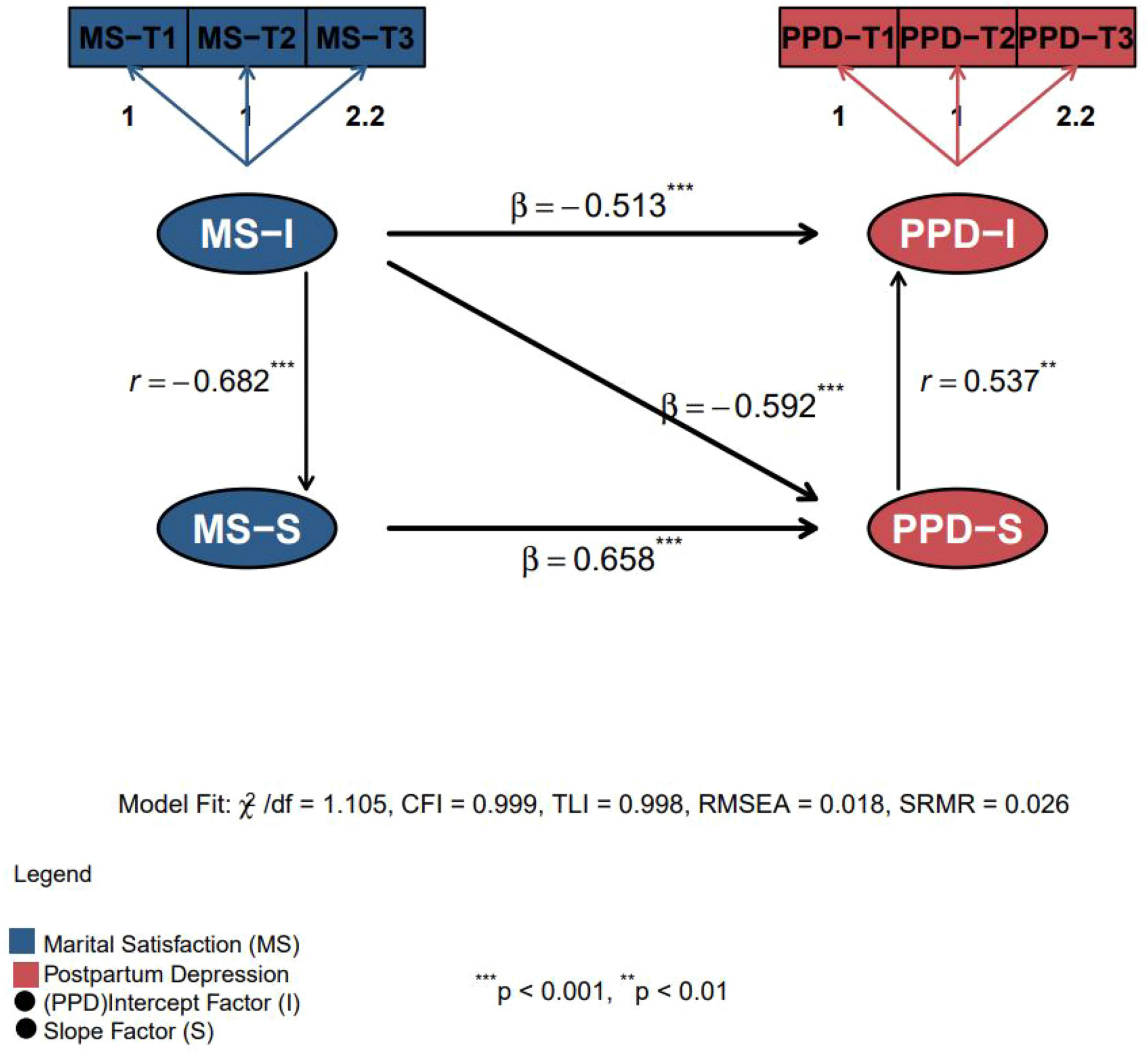
Latent growth model of parallel latent variable development patterns of marital satisfaction and postpartum depression in new mothers.

A parallel process latent growth model incorporating both marital satisfaction and postpartum depression was constructed (Figure 3). The model demonstrated excellent fit: χ^2^/df = 1.105, CFI = 0.999, TLI = 0.998, RMSEA = 0.018, SRMR = 0.026. The key findings are as follows: (1) The intercept of marital satisfaction significantly and negatively predicted both the intercept (β = –0.513, *P* < 0.001) and the slope (β = –0.592, *P* < 0.001) of postpartum depression. This indicates that a higher initial level of marital satisfaction was associated with a lower initial level of depression and a slower rate of increase in depressive symptoms. (2) The slope of marital satisfaction significantly and positively predicted the slope of postpartum depression (β = 0.658, *P* < 0.001), meaning that a faster decline in marital satisfaction was associated with a faster increase in depressive symptoms. (3) The intercept and slope of marital satisfaction were significantly negatively correlated (*r* = –0.682, *P* < 0.001), confirming that a higher initial level predicted a steeper decline. (4) The intercept and slope of postpartum depression were significantly positively correlated (*r* = 0.537, *P* = 0.008), indicating that a higher initial level of depression predicted a faster subsequent increase.

### 3.5 Cross-lagged regression analysis of marital satisfaction and postpartum depression

To examine the direction of the longitudinal association between marital satisfaction and postpartum depression, this study constructed and compared four nested cross-lagged models, controlling for potential confounding factors. To account for the influence of potential confounders, covariates including age, education level, occupation status, monthly household income per capita, mode of delivery, and infant sex were included in all models. These covariates were allowed to predict the observed variables of marital satisfaction and postpartum depression at each time point. Under the condition of controlling for the aforementioned covariates, the models were specified as follows: M1 (baseline model) included only the autoregressive paths for marital satisfaction and postpartum depression; M2 added the cross-lagged paths from marital satisfaction to subsequent postpartum depression to M1; M3 added the cross-lagged paths from postpartum depression to subsequent marital satisfaction to M1; M4 (full model) included all autoregressive paths along with bidirectional cross-lagged paths. The models were fitted using the maximum likelihood robust estimator, and model comparisons were conducted based on model fit indices and likelihood ratio tests. Model fit comparison results (Table 3) showed that the full model (M4) with bidirectional paths retained the best fit indices even after controlling for covariates. Specifically, M4 had the smallest χ^2^/df value, the highest CFI and TLI indices, and the lowest RMSEA and SRMR values. Further nested model likelihood ratio tests indicated that the fit of M4 was significantly better than that of the models containing only unidirectional paths (M2 and M3) (all Δχ^2^ values were significant). Therefore, M4 with covariates controlled was determined to be the optimal model representing the statistical association between the variables in this analysis.

**TABLE 3:** Comparison of cross-lagged model fit indices.

Model	Model specification	χ^2^(df)	CFI	TLI	RMSEA	SRMR	Model comparison	χ^2^(Δ df)
M1	Autoregressive (baseline)	167.85(54)	0.945	0.902	0.089	0.063	–	–
M2	M1 + paths from MS to PPD	73.21(52)	0.988	0.982	0.036	0.032	vs. M1	98.43(2)**
M3	M1 + paths from PPD to MS	153.28(52)	0.951	0.898	0.092	0.058	vs. M1	15.29(2)**
M4	Full model (all paths)	**60.15**(50)	**0.992**	**0.993**	**0.018**	**0.025**	**vs. M1**	**113.62**(4)**
							M2 vs. M4	15.18(2)**
							M3 vs. M4	98.32(2)**

**P < 0.001. Bold represents the final determined model.

**FIGURE 4:**
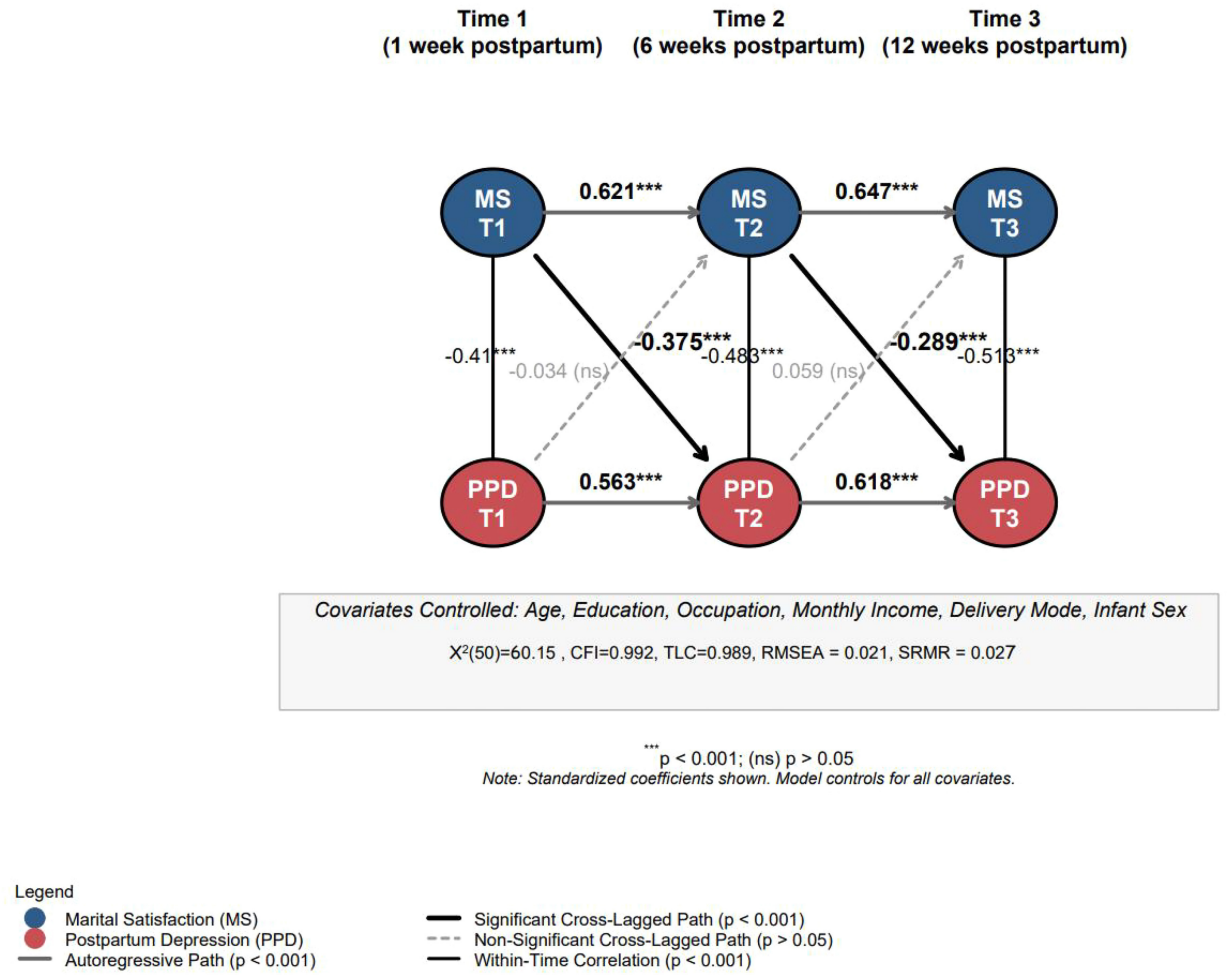
Cross-lagged model of marital satisfaction of postpartum women and postpartum depression (M4).

Based on the results of the optimal model with covariates controlled (path coefficients shown in Figure 4), a stable longitudinal association was observed where earlier marital satisfaction was linked to later postpartum depression levels: T1 marital satisfaction was significantly and negatively associated with T2 postpartum depression (β = –0.375, *P* < 0.001), and T2 marital satisfaction was also significantly and negatively associated with T3 postpartum depression (β = –0.289, *P* < 0.001). However, the corresponding cross-lagged paths in the reverse direction were not significant: T1 postpartum depression was not associated with T2 marital satisfaction (β = –0.034, *P* = 0.436), and T2 postpartum depression was not associated with T3 marital satisfaction (β = 0.059, *P* = 0.228).

These results are consistent with a unidirectional pattern of association from marital satisfaction to postpartum depression: after controlling for demographic and obstetric factors, the data indicate that marital satisfaction is longitudinally linked to subsequent depressive symptoms, whereas the reverse temporal association was not supported in this study.

### 3.6 Analysis of the moderating effects of demographic and obstetric factors

To preliminarily explore the boundary conditions of the association between marital satisfaction and postpartum depressive symptoms, this study employed hierarchical linear modeling, focusing on the “T1 Marital Satisfaction → T2 Postpartum Depression” association, which holds significant clinical relevance in the early postpartum period. The examination systematically investigated the moderating effects of demographic and obstetric factors. The selection of this specific path was primarily based on the following considerations: First, from a clinical practice standpoint, the 1–6 weeks postpartum period represents a critical window for identifying psychological risk and implementing early intervention; clarifying the moderating patterns of the association during this stage holds the greatest practical significance. Second, on a methodological level, the moderating variables examined (e.g., occupation, income, mode of delivery) are mostly relatively stable baseline characteristics; their statistical moderating effects are more direct in the early path and less susceptible to interference from other confounding factors. Third, for analytical focus, this approach avoids multiple comparisons across multiple time-point paths to control the risk of Type I error. Therefore, this section of the analysis is exploratory, aiming to preliminarily identify potential moderating variables. The results primarily apply to the interpretation of the early association path, and extrapolation to subsequent time points should be made with caution.

**FIGURE 5:**
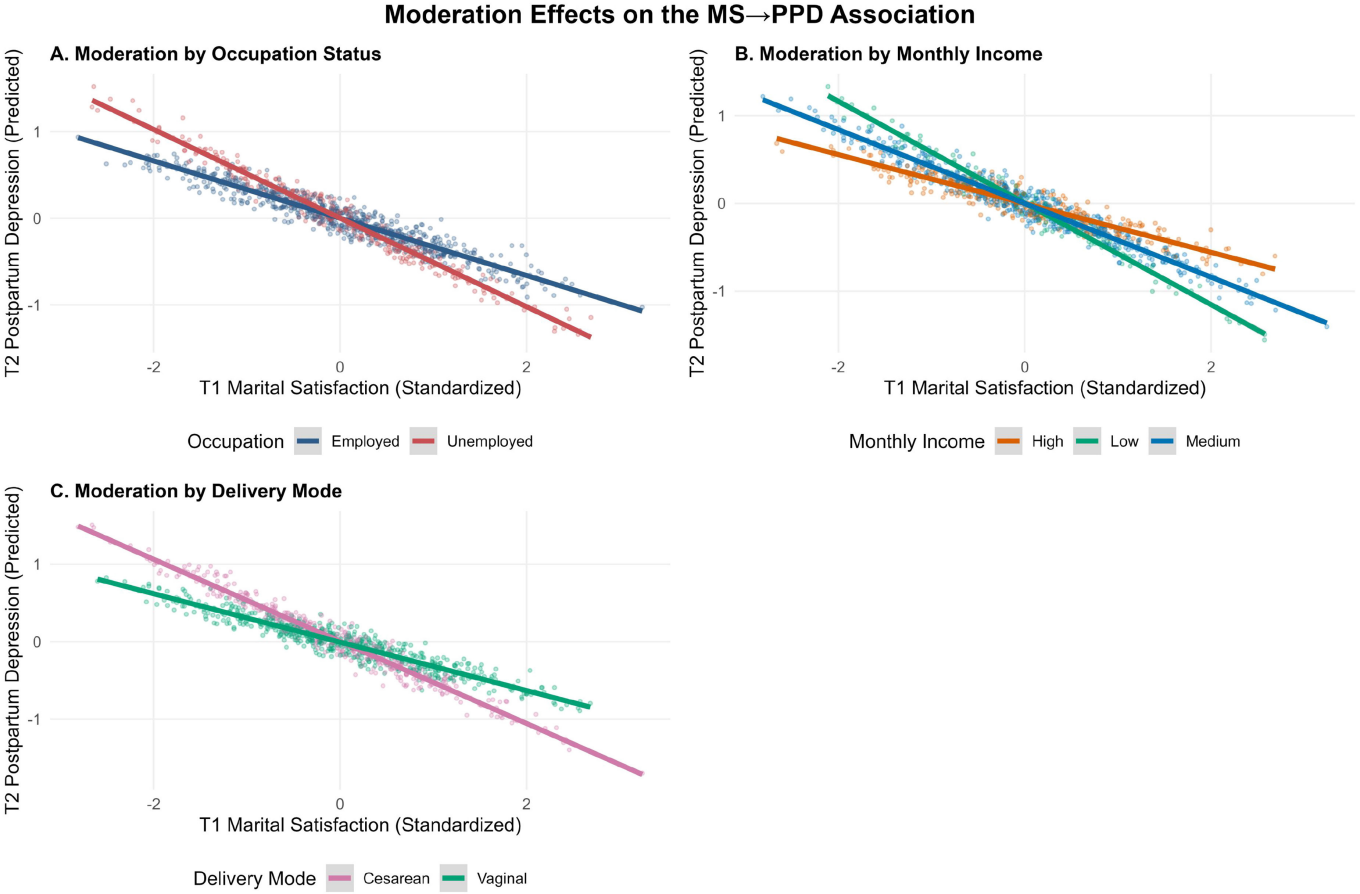
Analysis of the moderating effects of demographic and obstetric factors.

Based on the above design, we systematically tested the moderating effects of age, education level, occupation status, monthly household income per capita, mode of delivery, and infant sex on the target path. The results (see Table 4 and Figure 5) showed that occupation status, monthly household income, and mode of delivery had significant statistical moderating effects, whereas the moderating effects of age, education level, and infant sex were not significant. Specifically, the moderating effect of occupation status was significant (χ^2^ = 9.45, *P* = 0.002). The data indicated that the negative association between marital satisfaction and postpartum depression was more pronounced among unemployed/housewife women (β = –0.51) than among employed women (β = –0.33). The moderating effect of monthly household income was highly significant (χ^2^ = 12.37, *P* = 0.002) and displayed a clear dose-response pattern: the lower the income, the stronger the negative association (low-income group β = –0.58, high-income group β = –0.27). The moderating effect of mode of delivery was the strongest (χ^2^ = 15.83, *P* < 0.001). The negative association between the two variables was more pronounced among women who delivered via cesarean section (β = –0.53) than among those with vaginal delivery (β = –0.32).

**TABLE 4:** Analysis of the moderating effects of demographic and obstetric factors (*n* = 238).

Moderator and category	*n*	T1 MS→T2 PPD β (95% CI)	*P*-value	Between-group comparison
Occupation status				
Employed	156	−0.33 (−0.45, −0.21)	< 0.001	χ^2^(1) = 9.45, *p* = 0.002
Unemployed/Housewife	82	−0.51 (−0.67, −0.35)	< 0.001	
Monthly household income (RMB)				
< 5,000	31	−0.58 (−0.85, −0.31)	< 0.001	χ^2^(2) = 12.37, *p* = 0.002
5,000–10,000	127	−0.42 (−0.55, −0.29)	<0.001	
>10,000	80	−0.27 (−0.46, −0.08)	0.005	
Mode of delivery				
Vaginal delivery	138	−0.32 (−0.44, −0.20)	<0.001	χ^2^(1) = 15.83, *p* < 0.001
Cesarean section	100	−0.53 (−0.68, −0.38)	<0.001	
Education level				
High school/technical secondary school and below	65	−0.40 (−0.58, −0.22)	<0.001	χ^2^(2) = 2.15, *p* = 0.341
College/bachelor’s degree	160	−0.37 (−0.49, −0.25)	<0.001	
Master’s degree or above	13	−0.29 (−0.57, −0.01)	0.042	
Infant sex				
Male	122	−0.38 (−0.52, −0.24)	<0.001	χ^2^(1) = 0.21, *p* = 0.646
Female	116	−0.40 (−0.54, −0.26)	<0.001	
Age (years)*	238	−0.38 (−0.50, −0.26)	<0.001	χ^2^(1) = 0.87, *p* = 0.352

MS, Marital Satisfaction; PPD, Postpartum Depression; T1, 1 week postpartum; T2, 6 weeks postpartum. β coefficients are standardized. CI, confidence interval.

*Age is analyzed as a continuous moderator; the sample’s mean age was 28.71 ± 3.85 years.

## 4 Discussion

This study, through a 12-week longitudinal investigation, provided an in-depth exploration of the developmental trajectories of maternal marital satisfaction and postpartum depression and the dynamic relationship between them. The main findings revealed two distinct developmental patterns and clarified the longitudinal predictive association of marital satisfaction with postpartum depression, offering important empirical evidence for postpartum psychological interventions.

The results showed that marital satisfaction exhibited a linear decline trend during the postpartum period (slope = –0.69, *P* = 0.008), decreasing from 38.24 points at 1 week postpartum to 37.02 points at 12 weeks. This finding is consistent with the results of Bogdan et al., whose report indicated a declining trend in marital satisfaction over the first postpartum year (15). The decline may be attributed to several factors. First, increased parenting stress directly affects spousal interaction patterns (16), as the demands of newborn care reduce the couple’s alone time by 60–70%. Second, sleep deprivation acts as a mediating variable; reduced average daily sleep time in the first 3 months postpartum leads to decreased emotional regulation and conflict resolution abilities (17, 18). Furthermore, difficulties in adapting to role transitions are also a significant factor, especially for first-time mothers who face the dual challenge of adapting to the maternal role and redefining the spousal role simultaneously. On the other hand, postpartum depression showed a trend of initially increasing and then decreasing (*S* = 0.92, *P* = 0.005), peaking at 6 weeks postpartum (9.23 points) and slightly declining by 12 weeks (8.67 points). This trajectory is highly consistent with hormonal level changes (19, 20), where estrogen and progesterone drop to 1/100th of pre-pregnancy levels within the first week postpartum, directly affecting the function of the monoamine neurotransmitter system. Psychosocial factors are equally important; 6 weeks postpartum often marks a turning point where social attention diminishes, and the family’s focus shifts from the mother to the newborn, leading to feelings of loneliness and lack of support for the mother (21). Future research could consider employing quadratic growth models or more measurement time points to more precisely delineate the developmental trajectory of postpartum depression.

It is worth noting that the initial level of marital satisfaction showed a significant negative correlation with its rate of change (*r* = –0.682, *P* < 0.001), indicating that women with higher initial satisfaction experienced a faster rate of decline. This phenomenon may reflect a “high expectations-great disappointment” effect, where couples with initially high marital satisfaction had greater expectations for their postpartum relationship, leading to stronger feelings of disappointment when reality did not meet these expectations (22). Conversely, the initial level of postpartum depression showed no significant correlation with its rate of change (*r* = 0.508, *P* = 0.134), suggesting that regardless of the initial severity of depression, its pattern of change was relatively consistent.

Through the parallel process model, this study found that the initial level of marital satisfaction significantly predicted both the initial level (β = –0.513) and the rate of increase (β = –0.592) of postpartum depression. This indicates that a high-quality marital relationship may exert a protective effect through several mechanisms (23–25). First, it provides emotional support buffering, offering a safe space for emotional expression and reducing emotional suppression. Second, it involves practical assistance, such as sharing childcare and household burdens, which alleviates maternal stress. Finally, it fosters a sense of shared meaning, as couples face parenting challenges together, thereby strengthening the marital alliance.

The cross-lagged model analysis provided important longitudinal evidence regarding the temporal ordering of associations. Marital satisfaction had a stable negative predictive effect on postpartum depression (T1→T2: β = –0.382; T2→T3: β = –0.294), while the corresponding cross-lagged paths in the reverse direction were not significant. This finding is consistent with the theoretical perspective of marital satisfaction as a factor linked to postpartum depression (26) and aligns with the perspective of Family Systems Theory (27). which posits that marital relationship quality is associated with an individual’s resources for coping with stress and their emotion regulation ability, which in turn are related to susceptibility to depression. Regarding potential mechanisms, marital satisfaction may be linked to postpartum depression through several pathways: physiologically, a better marital relationship has been associated with promoted oxytocin secretion, inhibited excessive HPA axis activation, and lower cortisol levels (28); psychologically, marital satisfaction enhances self-efficacy and emotion regulation ability, reducing postpartum emotional fluctuations (29, 30). Notably, the strength of this longitudinal association for marital satisfaction weakened over time (from −0.382 to −0.294), which may reflect the increasing influence of other intervening factors. After 3 months postpartum, factors such as the quality of mother-infant interaction, individual adaptation capacity, and external support systems may have gradually exerted a stronger association with depression, relatively diminishing the explanatory contribution of marital satisfaction (31). This study also found that the strength of the concurrent correlation between marital satisfaction and postpartum depression increased over time, indicating that the relationship between the two became closer as the postpartum period progressed. This may be because the association of the marital relationship with mental health becomes more pronounced as parenting stress persists, or because both are linked to other factors whose associations intensify over time.

Analysis of the moderating effects in this study revealed that the protective effect of marital satisfaction on postpartum depression has clear boundary conditions, with its strength showing systematic variations depending on the personal and obstetric characteristics of the postpartum women. The study found that occupational status, monthly household income, and mode of delivery had significant moderating effects on the core predictive path. Specifically, the protective effect of marital satisfaction was stronger among unemployed or housewife women than among employed women. Among groups with different monthly household incomes, this protective effect showed a clear negative gradient, meaning it was stronger with lower income. Regarding the mode of delivery, women who underwent cesarean section derived significantly greater protective benefit from marital satisfaction than those who had vaginal deliveries. These results collectively suggest that when women are in situations with relatively limited resources or facing additional health challenges, their mental health status is more sensitive to and dependent on the quality of their marital relationship (32). On the other hand, factors such as age, education level, and infant sex did not show significant moderating effects. This indicates that the basic protective effect provided by marital satisfaction is relatively stable and universal across postpartum women with these different characteristics. These findings have direct value for guiding clinical practice. They suggest that in the screening and intervention work for postpartum mental health, the assessment and support of marital satisfaction should be prioritized and incorporated into the routine care processes for high-risk groups, such as unemployed/housewife women, those with low income, and those who have undergone cesarean section. For these populations, interventions focused on the couple’s relationship—such as enhancing communication skills, developing co-parenting plans, and providing specific postoperative collaborative guidance for families after cesarean section—may yield greater health benefits. Future intervention programs can be designed with greater refinement based on this to optimize resource allocation and maximize effectiveness.

The findings of this study hold significant theoretical implications and practical value. Theoretically, the research validates the applicability of family systems theory in postpartum psychological adaptation, clarifying the predictive role of marital satisfaction as a core family factor on postpartum depression. Simultaneously, the trajectory analysis provides a new perspective for understanding the dynamic process of postpartum psychological change, breaking through the static limitations of cross-sectional research. In terms of practical application, this study supports the implementation of a sequential intervention strategy. During the prenatal prevention stage, the focus should be on enhancing marital satisfaction through couple communication training, role expectation management, and conflict resolution skill training to strengthen marital resilience (33–35). Specific measures may include: ➀Prenatal couple workshops to provide realistic expectations about postpartum life; ➁Communication skills training, focusing on cultivating expression and listening abilities under stress; ➂Jointly developing a childcare division of labor plan to reduce postpartum role conflict. In the early postpartum period (1–6 weeks), dual monitoring and early intervention should be conducted: ➀Routine screening of marital satisfaction and depressive symptoms to identify high-risk couples; ➁Providing practical support, such as childcare assistance and household help, to reduce stressors; ➂Promoting emotional expression between spouses and encouraging joint participation in childcare activities. During the mid-postpartum period (7–12 weeks), the focus should shift to relationship repair and skill enhancement. For couples with a significant decline in marital satisfaction, short-term couple counseling should be provided. Parent support groups can be organized to facilitate experience sharing and social support building. Couples should be helped to redefine intimacy and adapt to the new marital dynamic within the context of parenting. Special attention should be paid to the group with initially high marital satisfaction. Although they start with a higher baseline, their rate of decline is faster, necessitating prevention of the “sharp decline from a high starting point” pattern.

This study has several limitations, which provide directions for future research. First, there are limitations in the study design and sample. The sample was drawn from a single medical center, and all data were based on maternal self-reports, which may affect the generalizability of the results. Although a longitudinal design was used, the 6-week intervals between the three measurements may not capture more subtle fluctuations in postpartum mood and relationship dynamics. Furthermore, this study did not consider the potential influence of cultural factors, as marital role expectations and the expression of depression may vary across cultural backgrounds.

Second, there are conceptual and methodological limitations at the measurement and instrument level. Firstly, marital satisfaction is inherently a dyadic construct, but this study collected reports only from the mothers. This may result in measurements that conflate the mother’s personal mood (particularly depressive symptoms) with the actual quality of partner interaction—a potential “emotional congruence bias”—which could inflate the statistical association between the variables. Secondly, the Edinburgh Postnatal Depression Scale (EPDS) used in this study is a screening tool; its results reflect the level of depressive symptoms, not a clinical diagnosis. This may lead to misclassification bias, meaning the association found in this study may differ in its mechanisms and strength from findings based on clinical diagnoses. Third, there are inherent limitations in the statistical modeling and the capacity for inference. Although this study employed advanced methods such as latent growth models and cross-lagged panel models, the validity of inference is constrained by model assumptions. A core limitation is that the traditional cross-lagged panel model cannot fully separate stable between-person differences (e.g., some mothers being consistently in a “high marital satisfaction–low depression” state due to stable traits) from true within-person dynamic changes. Consequently, the reported predictive paths may represent a mixed estimate of both. Additionally, although statistical tests did not indicate severe common method bias, the risk of residual shared method variance persists, as all variables originated from self-reports by the same participants. Fourth, there may be residual confounding due to unmeasured variables. Although the study controlled for a series of demographic and obstetric variables, several important known potential confounding factors for postpartum depression were not included, such as maternal sleep quality, infant temperament and health, breastfeeding status, and broader social support from sources other than the partner. These factors may be associated with marital satisfaction and independently related to depressive symptoms, potentially confounding the observed longitudinal association between the two. In summary, the findings of this study provide important preliminary longitudinal evidence for understanding the temporal and directional associations between marital satisfaction and depressive symptoms in the early postpartum period. However, given the above limitations, particularly concerning measurement specificity, model assumptions, and control for confounding, a cautious interpretation regarding causal inference is warranted. Future research could deepen exploration in this field by: ➀employing multi-center sampling and intensive multi-wave tracking designs; ➁integrating multi-source data, such as spousal reports, behavioral observations, and clinical diagnostic interviews; ➂using models that better separate within-person effects (e.g., the Random Intercept Cross-Lagged Panel Model); and ➃systematically incorporating a broader range of physiological, parenting, and socio-ecological covariates. Ultimately, evidence-based intervention programs targeting the couple’s relationship can be developed and validated on this foundation.

## 5 Conclusion

This study, through a longitudinal design, revealed the developmental trajectories and the interrelationship between marital satisfaction and depressive symptoms in the postpartum period. The main conclusions are as follows. First, marital satisfaction exhibited a linear declining trend, while postpartum depression showed an inverted U-shaped trajectory, indicating distinct developmental patterns for both within the first 12 weeks postpartum. Second, marital satisfaction demonstrated a stable longitudinal association with postpartum depression, which aligns with its theoretical positioning as a factor linked to depression risk. Finally, the data suggested a unidirectional pattern of association, specifically that marital satisfaction was longitudinally linked to postpartum depression, rather than the reverse. These findings underscore the potential importance of the marital relationship in postpartum mental health and provide an empirical foundation for interventions targeting the couple’s relationship to address postpartum depression. It is recommended that the assessment of marital satisfaction be incorporated into routine prenatal and postnatal check-ups to enable the early identification of high-risk groups. Enhancing marital quality through couple-focused interventions may ultimately help prevent and reduce the incidence of postpartum depression.

## Data Availability

The original contributions presented in this study are included in this article/supplementary material, further inquiries can be directed to the corresponding author.
